# Supplementary material for: Mesosphere of Carbon-Shelled Copper Nanoparticles with High Conductivity and Thermal Stability via Direct Carbonization of Polymer Soft Templates
Source: Materials (Basel). 2022 Oct 27;15(21):7536. doi: 10.3390/ma15217536 (PMC9654284; doi:10.3390/ma15217536)
Supplement: Supplementary file 1 [file materials-15-07536-s001.zip › materials-1855911-supplementary.pdf]

Supporting materials:

Mesosphere of Carbon-Shelled Copper Nanoparticles with High Conductivity and Thermal Stability via Direct Carbonization of Polymer Soft Templates

Min Huang [1,2], Xinyu Cao [2], Jingnan Zhang [2], Huiqun Liu [1,*], Jiaxin Lu [2], Danqing Yi [1] and Yongmei Ma [2,*]

[1] School of Material Science and Engineering, Central South University, Changsha 410083, China
[2] Institute of Chemistry, Chinese Academy of Sciences, Beijing 100190, China
* Correspondence: liuhuiqun@csu.edu.cn (H.L.); maym@iccas.ac.cn (Y.M.)

Experimental phenomena in preparation of organic polymer-wrapped copper nanoparticles (Cu@organic):

When Na-PNFS, PVP and $CuSO_4{\cdot}5H_2O$ were dissolved in water, a pale-yellow transparent solution was obtained (Fig. 1a). The solution with pale-yellow is related with Na-PNFS because PVP solution is colorless. We propose Na-PNFS and PVP can form polymer–surfactant complexes just like PVP and SDS. Upon the addition of NaOH solution, the liquid became green (Fig. 1b), resulting from the mixture of blue $Cu(OH)_2$ solution and pale-yellow solution. When the solution was added with NaOH (the pH value reached 10±0.2) and put into water bath, the color did not change further. The reduction agent $N_2H_4{\cdot}H_2O$ was added dropwise. When the temperature of the solution reached 50 °C, the color of the solution converted immediately to light green-yellow and became orange within a few minutes (Fig.1c). This indicated the formation of Cu_2O colloid [30, 31]. The thermostatic process was continued for another 90 min. The final reaction mixture was a uniform stable colloidal dispersion as shown in Fig.1d. The color changes indicated the formation of different organometallic complex particles. The product (e) was collected by centrifugation and dried which was reddish brown powder.

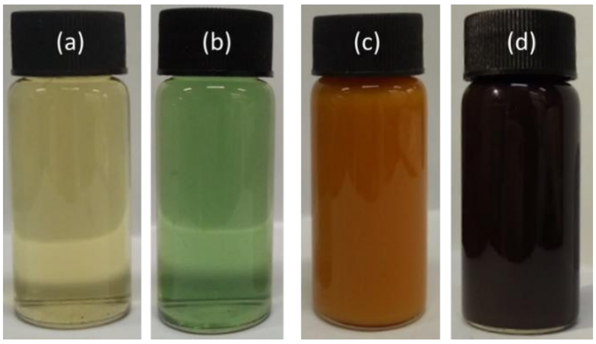

Figure S1. Photos of precursor samples (a) A-B-Cu^{2+} complex; (b) A-B-$Cu(OH)_2$ complex; (c) Cu_2O colloid; (d) Copper colloid.

Supplement discussion of the core-shell structure

Under high-magnification TEM micrograph (Figure S2), the shape of nanoparticle is not perfect spherical. The edges may relate to the absorption of surfactants which can controlling the particle morphology during reduction process.

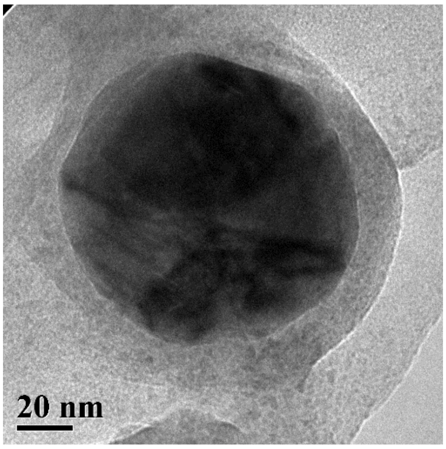

Figure S2. TEM image of Cu@organic.

The Cu@organic sample also showed excellent stability after exposing for 5 months at room temperature by XRD detection.

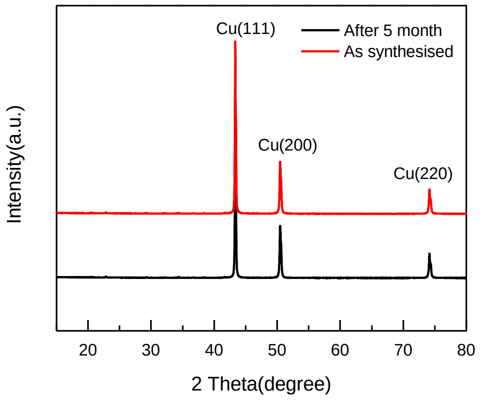

Figure S3. XRD patterns of Cu@organic (a) as synthesized; (b) stored in air at room temperature for 5 months.

Investigation of the component of Cu@organic and Cu@MC

EDX is a good method to measure element component for the bulk sample. And it can be used to analyze local region but not entire area. Actually, we did conduct EDX analysis, but the result only can show weight percent of Cu, S and O (Figure S4). The other light elements can't be measured accurately limited by the equipment.

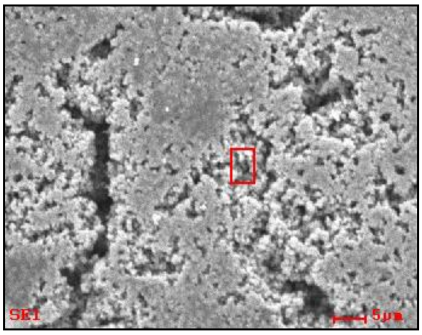

Element	*Wt%*	*At%*
OK	00.81	03.13
SK	00.79	01.51
CuK	98.40	95.36
Matrix	Correction	ZAF

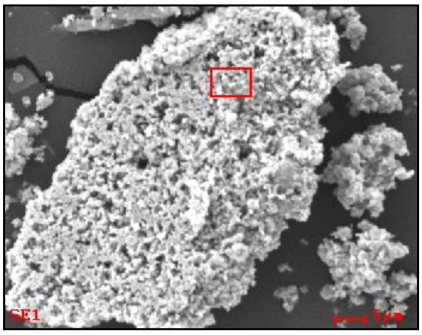

Element	*Wt%*	*At%*
OK	00.66	02.57
SK	00.78	01.50
CuK	98.56	95.94
Matrix	Correction	ZAF

Figure S4. SEM images of the Cu@organic (left) and SEDX measurement results (right).

In order to investigate the atomic weight percent of the Cu@organic materials, the carbon content in the Cu@MC was first estimated. As shown in Fig.9a, PVP is almost completely decomposed with only 2.3% remain at 750°C, this means the carbon layer on the copper surface is mainly derived from Na-PNFS. The TGA curve of Cu@MC (Fig.10b) shows that the final weight gain of Cu@MC at 800 °C is 23%. The complete oxidation of copper can result in a weight gain of 25% of copper, while the complete carbon oxidation can result in a weight loss of 100% of Carbon. The Carbon content or the loading amount of Cu in Cu@MC can be calculated from the actual weight gain and comparing with that of the theoretical pure copper. If the percentage of Cu is set as X in Cu@MC, the Carbon content% Y is 1-X. Assuming complete oxidation of copper and carbon occurred at 800°C, it can be deduced that the ratio of Cu/C is ~99/1. Figure 9a shows that the weight loss of Na-PNFs is about 50% at ≥800°C. These data suggest that the Na-PNFs content in Cu@organic is about 2wt%.

XPS analysis can give the element component information mostly of the surface. That is, it is more sensitive for the organic shell component. It indicates that the atom percent of the element of C, S, Cu, O, Na and N in Cu@organic material is 62.03%, 3.67%, 2.1%, 0.51%, 0.1% and 0.08%, respectively. From the atom ratio of S and N, the weight ratio of Na-PNF/ PVP can be estimated as 2/ 0.0199. So the weight percent of Cu, Na-PNFs and PVP in Cu@organic is around 98%, 1.98% and 0.02%.
